# Fatal Clostridium perfringens sepsis with spleen rupture and intraabdominal massive bleeding in a 37-week pregnancy

**DOI:** 10.1016/j.idcr.2021.e01355

**Published:** 2021-11-29

**Authors:** Evariste Gafumbegete, Berend Jacob van der Weide, Stefanie Misgeld, Henning Schmidt, Alaa Eldin Elsharkawy

**Affiliations:** a Department of Pathology, Ludmillenstift Hospital, Meppen, Germany; b Gynecology department, Hümmling Hospital Sögel, Sögel, Germany; c General surgery department, Hümmling Hospital Sögel, Sögel, Germany; d Neurosurgery Department, Bonifatius Hospital, Lingen, Germany

**Keywords:** Clostridium perfringens (CP), Sepsis, Spleen rupture, Intraabdominal massive bleeding, Pregnancy

## Abstract

The maternal death rate remains unacceptably high worldwide, predominantly in areas of poor access to quality health services. According to the WHO, in 2017, 810 women died from preventable causes related to pregnancy and childbirth. Causes of maternal death are plenty, including previous morbidity and unexpected causes. Among the latter are infectious disease-related deaths. Herein, we describe a case of a 29-year-old woman at 37 weeks’ gestation who presented with right upper quadrant pain, which was initially considered to be pregnancy-related. However, she collapsed shortly after the hospital admission. The physical examination revealed severe hypovolemic shock due to a large amount of intraperitoneal free fluid. The patient was immediately rushed into an emergency cesarean section followed by exploratory laparotomy, which demonstrated a large intra-abdominal hemorrhage. The patient and her fetus died in the operating room. An autopsy revealed acute gangrenous cholecystitis along with abundant rod-shaped bacteria within the mucosa and vessels of the gallbladder, gas gangrene and rupture of the spleen, and signs of shock.

Clostridium perfringens (CP) was isolated in the culture of a splenic sample. Although CP is a well-known and dreadful infectious etiological agent, catastrophic cases still happen. The acquaintance of this infection by the caregivers is crucial for the early diagnosis and treatment. This is a quite unique way to provide a dismal chance of survival in sepsis cases by this agent.

## Introduction

Advances in medical care have significantly reduced the risk of death during pregnancy and after delivery. Infection, pulmonary embolism, preeclampsia, and eclampsia are the most common causes of death of pregnant women despite these progress, particularly in western countries [1]. Immunological and physiological changes may make pregnant women more susceptible to severe infectious diseases [2], [3].

Currently, infection is the leading cause of maternal death [4]. Most infections can be promptly diagnosed and treated. However, there are challenging situations in which infections are difficult to detect. In some cases, the diagnosis is established too late or even after death. Infection by Clostridium perfringens (CP) is rare and characterized by nonspecific symptoms and a rapid course. Pregnancy-associated CP septicemia has become even rarer after the legalization of abortion in most western countries. Fulminant CP toxic shock causes multiorgan failure within one hour of the initial presentation, leading to sudden death [4], [5], [6].

CP infection mostly manifests with nonspeciﬁc and vague symptoms and is therefore usually unsuspected. Additionally, the laboratory workup and the determination of the Clostridium species are burdensome. [6].

The death of a pregnant woman is a sad and enraging event for her family and medical staff, particularly when it occurs unexpectedly. A postmortem examination can help the medical team to unravel the cause of death and bring comfort to the family [7].

Herein, we present the case of a healthy young woman with a normal pregnancy and no previous medical history who died of a CP infection three hours after admission Identification of the cause of death was possible only after autopsy.

## Case presentation

A 29-year-old woman (gravida II, para I) at 37 weeks gestation presented with abdominal pain in the right upper quadrant without any other symptoms. Her medical and gestational history was unremarkable. Her husband reported having eaten dried dates 2 days before admission. The patient collapsed shortly after entering the emergency room. Upon initial examination, she had a non-palpable pulse and non-recordable blood pressure. The ultrasonography revealed a large amount of intraperitoneal free fluid. The initial diagnosis was hypovolemic hemorrhagic shock secondary to a pregnancy-related intraperitoneal hemorrhage. Hence, cardiopulmonary resuscitation was initiated, and an emergency cesarean section was indicated for saving the fetus and exploring the source of bleeding. Cefazolin 2 g was administered. During uterine incision, the patient suffered a cardiac arrest; thus, resuscitation efforts were continued intraoperatively. The newborn infant was hypotonic and unmoving (with an Apgar score of 0) and therefore received resuscitation immediately. The uterus was not the source of bleeding.

The surgeon immediately further explored the abdominal cavity and opened the abdomen up to the xiphoid process. This revealed a large hemorrhagic region between the spleen and stomach. Resuscitation efforts were unsuccessful, and both mother and infant died. Due to the unclear cause of death, an autopsy was performed. The newborn autopsy was not undertaken according to the family´s wishes.

### Autopsy findings

**Fig. 1:**
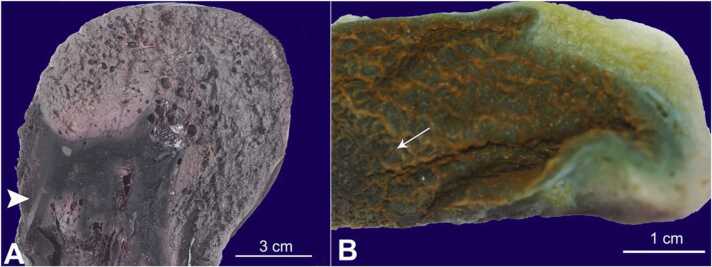
A - Gross view of the spleen. The arrowhead points a hematoma, and the remaining spleen shows gas-filled empty spaces. B - Gross view of the gallbladder: The arrow points the mucosal necrosis, and the remaining picture shows the normal gallbladder wall.

On autopsy, approximately three liters of clotted and unclotted blood collected in the peritoneal cavity. The spleen was perforated (3 cm in diameter, extending toward the pancreatic tail, along with a tear in the splenic artery. The splenic capsule was tense under pressure with gas inside (Fig. 1). A noticeable crackling sound could be heard upon palpation of the spleen. Gas was observed throughout the parenchyma of the spleen. Gas was also detected in the retroperitoneal adipose tissue along the abdominal and thoracic aorta. Moreover, a few non-adhered free blood clots were found in the heart ventricles with a subendocardial hemorrhage.

### Microscopic and histological findings

**Fig. 2:**
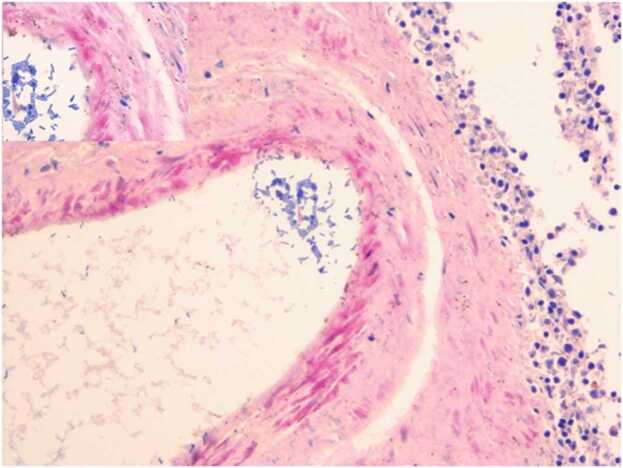
– Photomicrograph of the spleen with CP colonies are surrounded by gas in the splenic parenchyma and in the splenic vein and parenchyma. The inset (in the upper left) shows a detail of the colonies. (H&E, 200X).

**Fig. 3:**
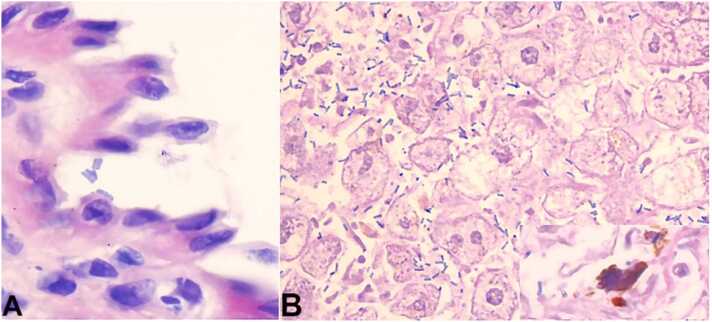
– Photomicrographs of: A – Vaginal wall. Vaginal squamous mucosa infected with Clostridium perfringens (HE, 100×), B - Liver parenchyma. Clostridium perfringens rods producing gas in the ductuli between the hepatocytes and inducing the gall thrombi in the ductuli at the bottom right. HE, 100 x.

**Fig. 4:**
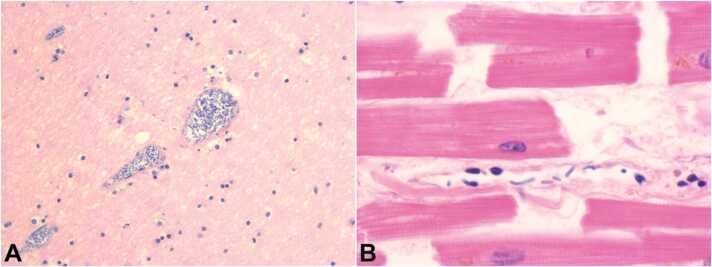
– A Photomicrography of the cerebellum with capillaries infected with CP (H&E, 60X); B – Photomicrograph of the myocardium with capillaries infected with CP (H&E, 200X).

Significant numbers of bacilliform bacteria were found in the spleen (Fig. 2), gallbladder mucosa, blood vessels; vagina (Fig. 3A) Liver parenchyma (Fig. 3B), cerebral, cerebellum capillaries and myocardium (Fig. 4A and 4B); perihilar lymph nodes; colonic crypts; renal and arteries.

There were signs of shock, interalveolar edema, necrosis, and fibrinous secretions in the lungs. We also noticed coagulative necrosis of the pancreas and stomach, swelling of the renal tubules, and acute gangrenous cholecystitis. Additionally, the gas formation was observed in the perivascular and intramuscular parts of the heart, spleen, kidney, vagina and cervical lips. Vaginal examination showed extensive abrasion of the mucosa with a white coating and exfoliated squamous epithelium; only the basal cell layer was still attached. Absolute absence of neutrophilic infiltration was noticed in histological findings. Other organs appeared normal.

### Microbiological findings

The culture was negative in aerobiosis. However, the incubation of the spleen sample in anaerobiosis with Schaedler Agar with 5% sheep blood-streaked at 35 degrees, isolated CP after 48 h. The identification was carried out using Maldi-TOF MS. The antibiogram was read according to EUCAST. Sensitive to penicillin, amoxicillin/clavulanic acid, piperacillin/tazobactam, and clindamycin.

Based on the gross findings at autopsy, histopathological examination, and laboratory results, we concluded that the patient died of hemorrhagic shock due to splenic rupture caused by CP sepsis (gas gangrene).

## Discussion

CP is a rod-shaped, spore-forming, gram-positive, anaerobic bacterium; it is one of the most widespread bacteria in the environment and is found in food, soil, spoiled vegetables and plants, marine fruits, intestines of vertebrates, insects, and stool [8].

Not only does CP constitute a part of the normal flora of the human and animal intestines, but it is also normally found in the vagina of approximately 10% of healthy women; however, a small number of women hosting this bacterium develop CP infection [8], [9].

Approximately 640 different species of Clostridium are known, of which only 5% are virulent [10].

CP doubles every 7 min [5] and produces approximately 20 toxins, of which the most harmful type is an alpha-toxin. This toxin causes intravascular hemolysis and anaerobic fermentation, thus producing large amounts of hydrogen sulfide, a gaseous waste product that could be the key determinant of mechanical tissue injury [11], [12]. Alpha and theta toxins decrease cardiac output, heart rate, and systemic vascular resistance [13]. CP endotoxin damaged the endothelium of the vessels and membranes of the red blood cells, which subsequently led to intravascular hemolysis, which in turn exaggerate the anaerobic environment that enhances the toxin production, developing a vicious circle and finally leading to toxic shock [5].

Clinical presentation of CP varies widely from non-specific without fever to septic shock. Most infections are transient contamination and may cure with antibiotics [10]. CP is the leading cause of traumatic gas gangrene, food poisoning, antibiotic-associated diarrhea, and enteritis necroticans [6], [14].

In some cases, CP infection may be presented with anemia, hemoglobinuria, and hemoglobinemia [12]. In the case of sepsis typical presentations are hemolytic anemia, renal failure, jaundice, hemoglobinuria, and gas gangrene [9], [8].

According to a Canadian report, CP causes septicemia in 0.7 cases per 100,000 population per year in the community [15].

The most common causes of CP septicemia are (i) food intoxication, (ii) wound contamination, and (iii) dissemination from the urogenital or gastrointestinal tracts in patients with malignancies or immunodeficiency. CP grows and spreads throughout the body under anaerobic conditions, with its incubation period varying between 6 and 72 h [8]. Cases of CP septicemia without an evident infectious focus have also been reported in the literature. CP septicemia presents a high mortality rate (70–100%) [16].

It is a generally challenging diagnosis in pregnant women. The lack of symptoms and specific signs as well as the low prevalence of this infection can lead to incorrect or delayed diagnosis [11].

Another factor that poses a threat to the survival of patients with CP infection is time, i.e., these patients may survive only if their disease is promptly diagnosed and rapidly treated. Unfortunately, in our case, the time from admission to death was 3 h, and the accurate diagnosis could be established only after autopsy. According to previous studies, fetal septicemia can occur within 12–24 h from the onset of infection [10].

In our case, CP infection originated from a necrotic gallbladder (an anaerobic environment) in the setting of pregnancy-related disturbances of the immune system. The patient had eaten dried dates two days before admission, which may be the source of infection; unfortunately, we do not have a sample to test. Food has been reported as the source of infection.

The history of colic pain in the right upper quadrant of the abdomen 2 days before death and the necrotic gallbladder at autopsy supported our hypothesis. Moreover, the hepatobiliary system (45.0%) has been reported as the most common origin of CP infection, followed by invasive intestinal or gynecological procedures [17].

In agreement with our case, Pun and Wehner [18] have reported a similar case of a patient with abdominal pain and emphysematous cholecystitis who died from sepsis and intravascular hemolysis. The vaginal lesions may also be another source of septicemia; since it showed a significant site of infection with CP depicted in the autopsy. The clinical history and lack of infection in the uterus rule it out as a source of infection with CP.

Intra-abdominal bleeding due to splenic rupture was the underlying cause of death in our patient. Nontraumatic splenic rupture may occur in several conditions, including (i) increased intrasplenic tension, (ii) compression of a diseased spleen, and (iii) vascular occlusion [19]. In our case, all these mechanisms acted together and consequently increased intrasplenic pressure, hemolysis, vascular damage, and colic pain [11].

In cases like ours where the cause of death is unclear, an autopsy is essential to ascertain the cause of death and prevent similar tragedies from happening in the future The autopsy of our patient revealed the cause of death and the related extensive pathologic changes [7]. This autopsy helped our gynecological team and provided us with valuable information.

The cornerstones of management are early recognition and aggressive treatment with broad-spectrum antibiotics and surgical debridement; hyperbaric oxygen (up to 2 bar 100% pure oxygen) also proves to be useful [20].

## Conclusion

The current report highlights that *C. perfringens* sepsis remains a dangerous and lethal condition that can occur anytime. Caregivers and physicians should be familiar with this rare, fatal infection. Pathologists and clinicians should work together to determine the cause of death in unclear cases.

This study was carried out at Ludmillenstift Hospital – teaching Hospital of Carl-von-Ossietzky-University, Oldenburg, Department of Pathology. Meppen, Germany.

## Ethics approval and consent to participate

The authors retain informed consent signed by the deceased’s next of kin, and the manuscript is approved/exempted by the Institutional Ethics Committee.

## Financial support

The authors declare that no financial support was received.

## Author Contributions

Evariste Gafumbegete was responsible for study conception design, acquisition of data, drafting of manuscript and design the figures. Berend Jacob van der Weide did the analysis and interpretation of data. Stefanie Misgeld contributed to acquisition of data and Critical revision. Henning Schmidt provided critical feedback and helped shape the research, and Critical revision. Alaa Eldin Elsharkawy wrote the final form of the manuscript, shaped the research and supervised the research.

## Conflict of interest

The authors have no conflict of interest to declare.
